# A Facile Method for Loading CeO_2_ Nanoparticles on Anodic TiO_2_ Nanotube Arrays

**DOI:** 10.1186/s11671-018-2504-7

**Published:** 2018-04-03

**Authors:** Yulong Liao, Botao Yuan, Dainan Zhang, Xiaoyi Wang, Yuanxun Li, Qiye Wen, Huaiwu Zhang, Zhiyong Zhong

**Affiliations:** 1 State Key Laboratory of Electronic Thin Film and Integrated Devices, University of Electronic Science and Technology of China, Chengdu, 610054 China; 2 Center for Applied Chemistry, University of Electronic Science and Technology of China, Chengdu, 610054 China

**Keywords:** Anodic TiO_2_ nanotubes, CeO_2_ nanoparticles, CeO_2_/TiO_2_ heterojunctions, Green chemistry

## Abstract

In this paper, a facile method was proposed to load CeO_2_ nanoparticles (NPs) on anodic TiO_2_ nanotube (NT) arrays, which leads to a formation of CeO_2_/TiO_2_ heterojunctions. Highly ordered anatase phase TiO_2_ NT arrays were fabricated by using anodic oxidation method, then these individual TiO_2_ NTs were used as tiny “nano-containers” to load a small amount of Ce(NO_3_)_3_ solutions. The loaded anodic TiO_2_ NTs were baked and heated to a high temperature of 450 °C, under which the Ce(NO_3_)_3_ would be thermally decomposed inside those nano-containers. After the thermal decomposition of Ce(NO_3_)_3_, cubic crystal CeO_2_ NPs were obtained and successfully loaded into the anodic TiO_2_ NT arrays. The prepared CeO_2_/TiO_2_ heterojunction structures were characterized by a variety of analytical technologies, including XRD, SEM, and Raman spectra. This study provides a facile approach to prepare CeO_2_/TiO_2_ films, which could be very useful for environmental and energy-related areas.

## Background

As is well known, titanium dioxide (TiO_2_) materials have been widely used for a great number of applications such as solar cells, water treatment materials, catalysts and so on [1–6]. The reason for TiO_2_ and TiO_2_-derived materials have so many applications is they have outstanding photocatalytic, electrical, mechanical, and thermal properties [7–9]. In nature, TiO_2_ has three most commonly encountered crystalline polymorphs, including anatase, rutile, and brookite. Among the three TiO_2_ polymorphs, anatase is the most photoactive polymorph used for degradation of organic pollutants or electrodes for energy applications [10, 11]. Anatase TiO_2_ have a band gap of ~ 3.2 eV, and it has shown good catalytic activity, corrosion resistance, and light resistance. Alone with its stable performance, low cost, non-toxic harmless, TiO_2_ in anatase phase was recognized as the best photocatalyst.

**Fig. 1:**
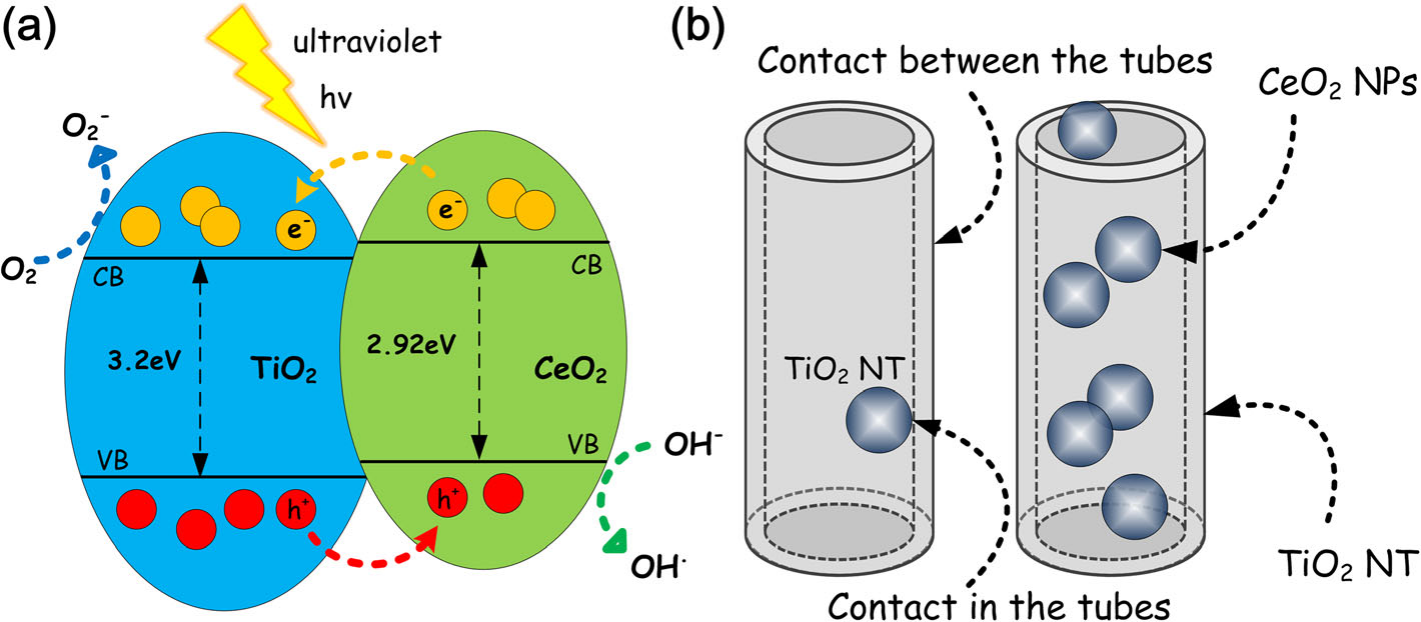
**a** Energy levels of TiO_2_ NTs and CeO_2_ NPs with electron-hole pair transfer and separation. **b** Illustration diagram of CeO_2_ NP and TiO_2_ NT heterojunction

Recently, TiO_2_ nanotube (NT) arrays have attracted great attention due to its unique tubular structure-induced advantages [12–18]. However, their performances were still limited by inherent material faults, such as relatively wide gaps (~ 3.2 eV) [19–22]. In order to achieve better application, narrow band semiconductors with proper energy level were proposed to further modify TiO_2_ NT arrays [23, 24]. The band gap of cubic CeO_2_ is about 2.92 eV and has good chemical stability. TiO_2_ modified by CeO_2_ were found very useful in the field of photocatalysis, gas sensors, and so on [25–27]. In the field of photocatalysis, the rapid recombination of photogenerated electron-hole pairs of reduces the photocatalytic performance of TiO_2_. However, the modification of CeO_2_ changes the recombination rate of the electron-hole pairs inside a CeO_2_/TiO_2_ composite material. As shown in the Fig. 1a, once CeO_2_/TiO_2_ heterojunctions are formed, more superoxide and hydroxyl radicals could be produced, leading to improved photocatalytic performance. In the field of gas sensors, CeO_2_ is a promising material for oxygen gas sensing at high temperature. TiO_2_ modified by CeO_2_ could effectively improve the adaptability of gas sensor, because the CeO_2_/TiO_2_ heterostructures enable the sensing of oxygen gas at low operating temperatures (< 500 °C) [28]. In order to prepare CeO_2_/TiO_2_ heterostructures, many approaches have been proposed including sol-gel method and hydrothermal method [29–31]. The former works were found very interesting and their products had shown good performances. However, the traditional methods are always used to prepare CeO_2_/TiO_2_ heterostructures in powder form and often with complicated procedures. For preparing CeO_2_/TiO_2_ heterostructures based on TiO_2_ NTs as shown in Fig. 1b, developing facile method to load CeO_2_ nanoparticles (NPs) on the TiO_2_ NT arrays is highly desired. To this end, we proposed a novel method for the preparation of CeO_2_/TiO_2_ heterojunctions in this study.

Highly ordered anatase phase TiO_2_ NT arrays were fabricated by anodic oxidation method, then the individual TiO_2_ NTs were prepared as tiny “nano-containers” to load Ce(NO_3_)_3_ solutions. The loaded anodic TiO_2_ NTs were heated to a high temperature, under which the Ce(NO_3_)_3_ were thermal decomposed. After the thermal decomposition of Ce(NO_3_)_3_, cubic crystal CeO_2_ NPs were obtained and successfully loaded into the anodic TiO_2_ NT arrays. CeO_2_/TiO_2_ heterojunctions prepared by this method was recognized as simple operation, low cost, non-toxic harmless.

## Experimental Section

### Synthesis of TiO_2_ Nanotube Arrays

Firstly, we used anodic oxidation method to prepare TiO_2_ nanotube arrays [32–34]. Briefly, titanium pieces were cut into small pieces (5 cm × 1.5 cm) and flattened. After being washed in detergent water, the titanium pieces were washed in an ultrasonic cleaner for 1 h with deionized water and alcohol, respectively. The dried titanium sheets with a counter electrode were immersed in the prepared electrolyte (500 ml glycol, 10 ml H_2_O and 1.66 g NH_4_F) under room temperature. A constant voltage of 60 V was applied to the two electrodes for 2 h. Then, TiO_2_ NT films were annealed at 450 °C for 3 h, and the rate of anatase TiO_2_ NTs were obtained.

### Synthesis of CeO_2_/TiO_2_ Heterojunction

**Fig. 2:**
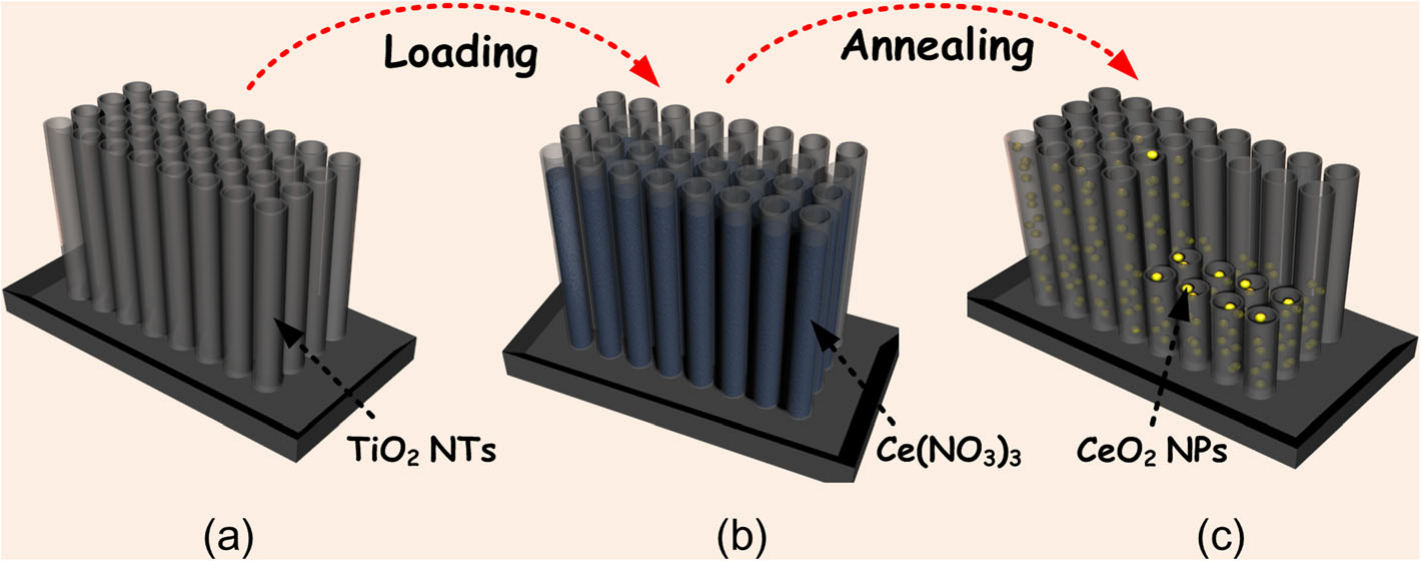
Synthesis flow of CeO_2_/TiO_2_ heterojunction: (a) preparation of empty TiO_2_ NTs, (b) loading the TiO_2_ NTs with Ce(NO_3_)_3_ solution, and (c) formation of CeO_2_/TiO_2_ heterojunction structures

The individual TiO_2_ NTs inside the anodic films were taken as thousands small nano-containers to load the raw materials of CeO_2_, which will be full with the Ce contained solutions. As shown in Fig. 2, the TiO_2_ NTs were immersed in the Ce(NO_3_)_3_ solution (concentration were 0.05, 0.1, 0.2,0.5, and 1 mol/L respectively) for 3 s. In order to ensure the open tube mouth of the TiO_2_ NTs, it is worthy of attention that superfluous solution on the surface of the TiO_2_ NT films should be absorbed by using a qualitative filter paper immediately. The films were tilted as much as possible, making the solution flow to the edge of the films, and the filter paper was used to dry out the superfluous solution to ensure uniformity of solution. Then, the loaded films were dried at 70 °C for 1 h, during which the Ce(NO_3_)_3_ solute will be deposited inside the TiO_2_ NT nano-containers. And the dried films were further annealed at 450 °C for 2 h, during which the deposited Ce(NO_3_)_3_ will be thermally decomposed into CeO_2_ NPs at a high temperature. Finally, CeO_2_ NPs were obtained and attached to each single TiO_2_ NT of the arrays.

### Characterization

Crystalline structure of the CeO_2_/TiO_2_ heterojunction was analyzed by X-ray diffraction (XRD; D/max 2400 X Series X-ray diffractometer). XRD was applied to characterize the samples at a step of 0.03° in the range of 10° to 80°. The microstructure of the heterojunctions and the morphology of the nanotubes were characterized by scanning electron microscopy (SEM; JSM-7000F, JEOL Inc. Japan). The elemental distribution of the microscopic region of the materials was qualitatively and quantitatively analyzed by energy-dispersive spectrometry (EDS). The crystal structure of the CeO_2_/TiO_2_ heterojunction was also analyzed by Raman spectra (inVia, Renishaw, UK). Resonant Raman scattering spectra were recorded at room temperature to obtain a more clear display of components.

## Results and Discussion

### Crystalline Properties of the Prepared CeO_2_/TiO_2_ Heterojunction Films

**Fig. 3:**
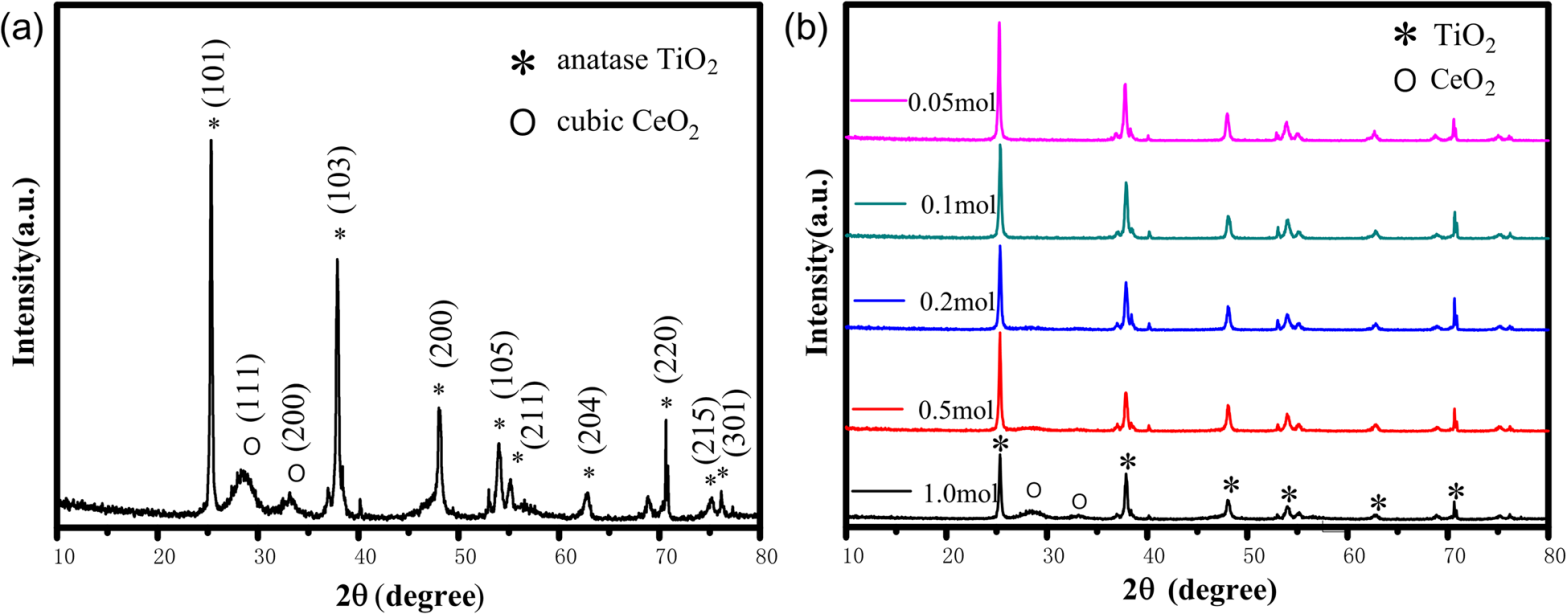
**a** XRD pattern of the anatase phase of TiO_2_ and cubic CeO_2_. **b** XRD pattern of the anatase phase of TiO_2_ and cubic CeO_2_ with different concentrations of Ce(NO_3_)_3_

XRD patterns of the prepared CeO_2_/TiO_2_ heterojunction films are shown in Fig. 3. The diffraction peak could be identified as the anatase phase of TiO_2_ and cubic phase of CeO_2_. The diffraction peaks located at 25.28°, 36.80°, 37.80°, 48.05°, 53.89°, 55.06°, 62.68°, 70.30°, 75.03°, and 76.02° were attributed to the anatase lattice plane (101), (103), (004), (200), (105), (211), (204), (220), (215), and (301), respectively. Moreover, the minor diffraction peaks at 40.1° and 53.0° were attributed to (101) and (102) of Ti (see Fig. 3a). This indicates the anodic TiO_2_ NT films have an anatase crystalline structure in this study. In the crystallization process, anatase grains usually have a smaller size and a larger specific surface area. Therefore, anatase TiO_2_ surface has strong adsorption capacity of H_2_O, O_2_, and OH^−^ and its photocatalytic activity is greatly high [35, 36]. The adsorption capacity of the anatase TiO_2_ NT films is enormously influenced in the photocatalytic reaction, and the strong adsorption capacity is beneficial to its activity. Meanwhile, the diffraction peak located at 28.55° and 33.08° was indexed to crystal face (111) and (200) of CeO_2_, respectively [37, 38]. Figure 3b shows the XRD patterns of the CeO_2_/TiO_2_ heterojunction films with different initial Ce(NO_3_)_3_ concentration. When the concentration of Ce(NO_3_)_3_ was too low, only diffraction peaks of the anatase TiO_2_ could be observed. With the concentration of Ce(NO_3_)_3_ gradually increasing, the cubic phase of cerium oxide appeared and the diffraction peaks of cubic CeO_2_ became stronger. According to the tested XRD data, the standard PDF showed CeO_2_ has a face-centered cubic (FCC) crystal structure. The calculated lattice parameters were *a* = *b* = *c* = 0.5411 nm and *α* = *β* = *γ* = 90°, which matched with the standard PDF. It could be summarized that TiO_2_ was modified by CeO_2_ perfectly in lattice matching so that their heterojunctions are tighter and better to produce a special electron transfer process which is able to facilitate the separation of the electron/hole pairs.

### Microscopic Morphologies of the CeO_2_/TiO_2_ Heterojunction Films

**Fig. 4:**
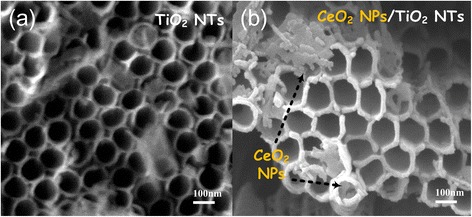
Typical SEM images of **a** pure TiO_2_ nanotube arrays without modification and **b** the CeO_2_/TiO_2_ heterojunction, indicating the highly ordered structure with open tube mouth morphology, and after modification, CeO_2_ was successfully loaded into the TiO_2_ nanotube arrays

**Fig. 5:**
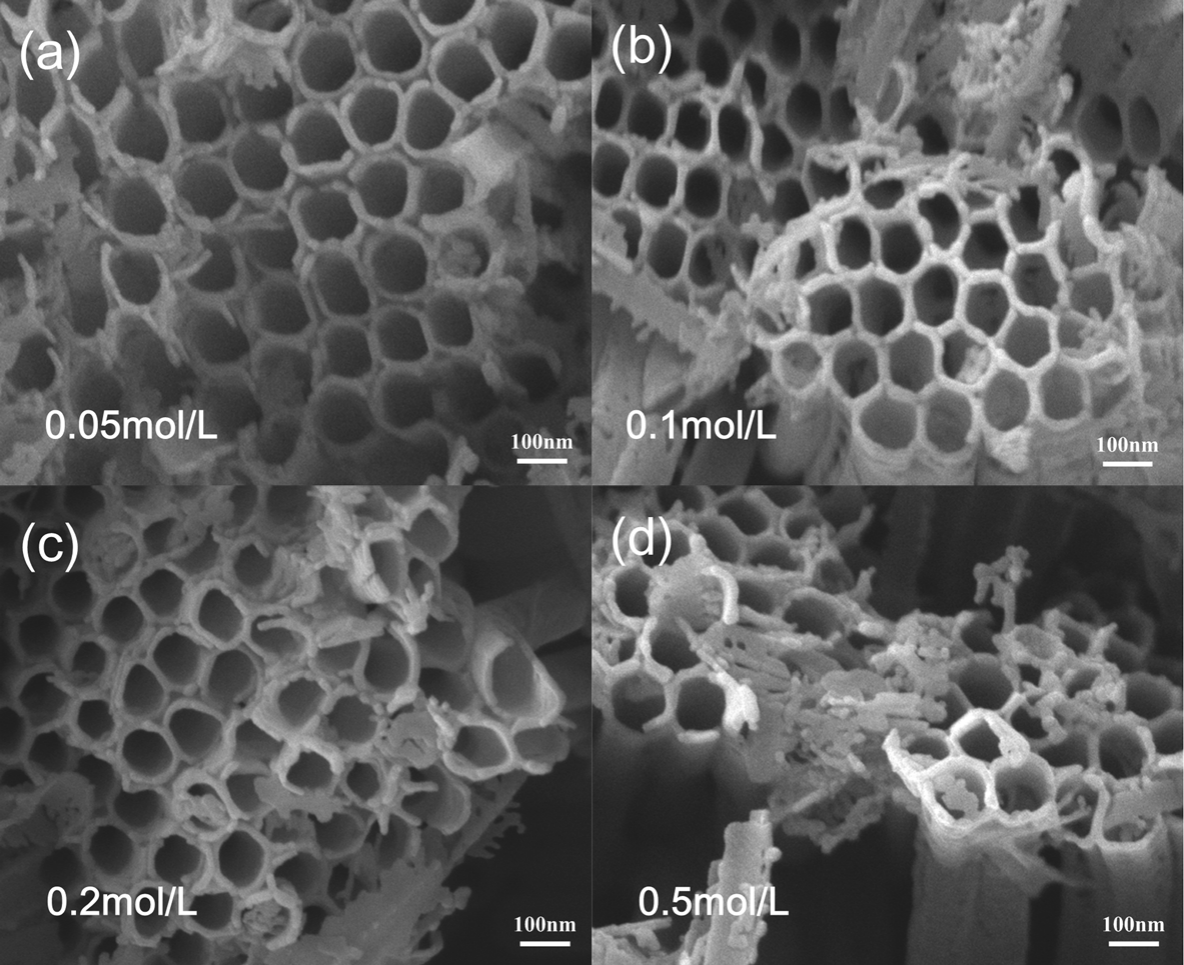
SEM images of the CeO_2_/TiO_2_ heterojunctions with different Ce(NO_3_)_3_ solution concentration: **a** sample immersed in 0.05 mol/L Ce(NO_3_)_3_; **b** sample immersed in 0.1 mol/L Ce(NO_3_)_3_; **c** sample immersed in 0.2 mol/L Ce(NO_3_)_3_; and **d** sample immersed in 0.5 mol/L Ce(NO_3_)_3_

Figure 4 shows SEM images of the anatase TiO_2_ nanotube arrays before and after being modified by CeO_2_. Top profile of the TiO_2_ NT arrays without loading CeO_2_ is shown as Fig. 4a, and the self-organized NT arrays were found quite dense and had an open-mouth top morphology, which provides a passage way for the Ce(NO_3_)_3_ solution filling into the NTs in this study. The average tube diameter is evaluated about 110 nm. Figure 4b shows the microstructure of anodic TiO_2_ NTs modified by CeO_2_ NPs. It can be seen that there are lots of long strips on the tube-pore mouths by comparing to the pure TiO_2_ NTs. Meanwhile, the tube wall thickness could be found getting increased by taking a close look. These observations indicate that the morphologies of the anodic TiO_2_ NT arrays have an obvious change after the loading and annealing process. Also, from the SEM images, most CeO_2_ NPs were deposited on the top of the TiO_2_ NTs, because when the superfluous Ce(NO_3_)_3_ solution was treated, the superfluous solution on the top of tubes was not completely disposed, and after thermally decomposed, the CeO_2_ NPs were deposited on the top of tubes. Morphologies of the CeO_2_/TiO_2_ heterojunction films with Ce(NO_3_)_3_ solution concentration varying from 0.05 mol to 0.5 mol are shown in Fig. 5. It could be clearly seen that with the Ce(NO_3_)_3_ solution concentration increasing, the nanoparticles in the TiO_2_ NTs gradually became more abundant and more elongated particles appeared on the TiO_2_ NTs. These results reveal that the CeO_2_ nanoparticles are successfully attached to tube wall of the anodic TiO_2_ NT arrays, forming a CeO_2_/TiO_2_ heterojunction structure. The large specific surface area of the TiO_2_ NTs provides a good substrate for CeO_2_ NPs to load onto the anodic TiO_2_ NT films.

### Components Analysis of the CeO_2_/TiO_2_ Heterojunction Films

**Fig. 6:**
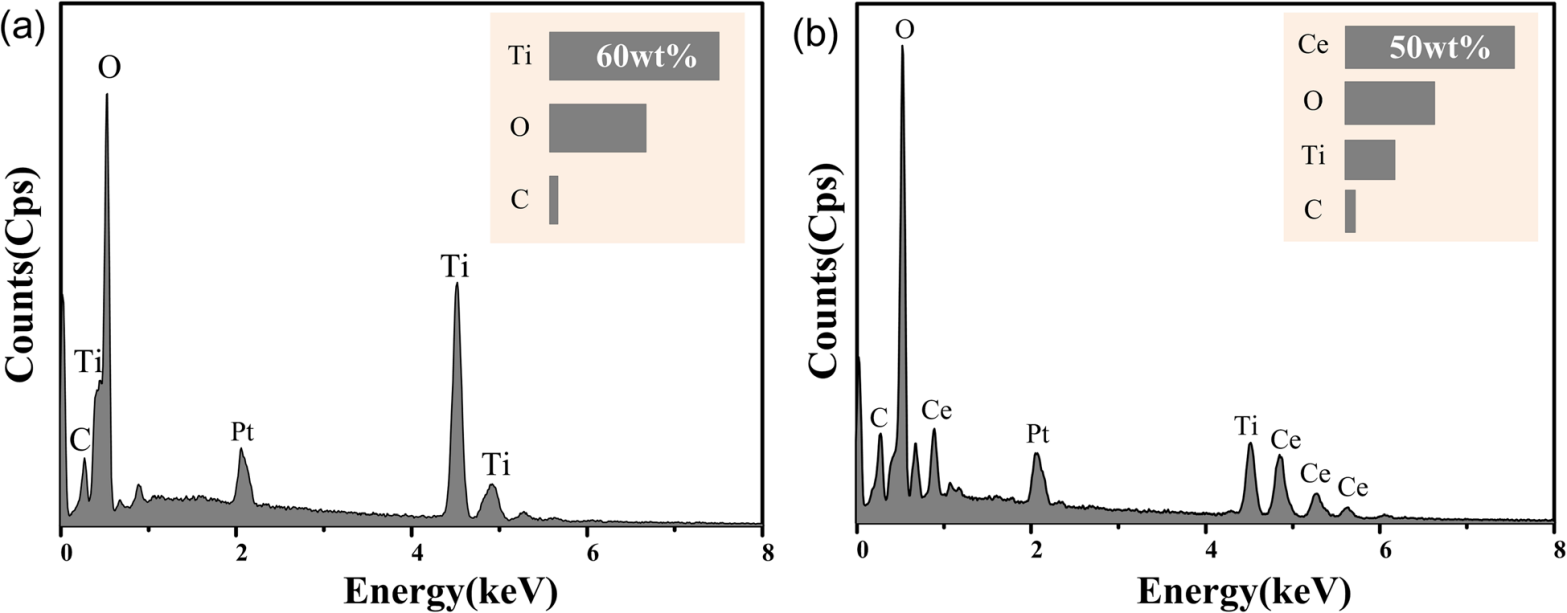
EDS results of **a** pure TiO_2_ NTs and **b** CeO_2_/TiO_2_ heterojunction, showing the existence of element Ti, Ce, and O after loading Ce(NO_3_)_3_. The results confirm the successful loading of CeO_2_ on the TiO_2_ NTAs

In order to coordinate with the SEM test results, energy-dispersive X-ray spectroscopy (EDS) was used to analyze the elemental composition of the CeO_2_/TiO_2_ heterojunction films. EDS comparison diagram between TiO_2_ NTs and CeO_2_/TiO_2_ heterojunction is shown in Fig. 6. As shown in the Fig. 6a, only Ti and O could be detected. The atomic percentage of Ti and O elements is 27.37 and 65.36%, respectively. The sample of CeO_2_/TiO_2_ heterojunction film which is prepared in the 0.1 mol/L Ce(NO_3_)_3_ solution is shown in Fig. 6b. Ce, O, and Ti could be detected. The atomic percentage of Ce, Ti, and O elements is 11.91, 12.04, and 59.98%, respectively. It can be concluded from the EDS results that CeO_2_ NPs were successfully deposited on the TiO_2_ NTs.

**Fig. 7:**
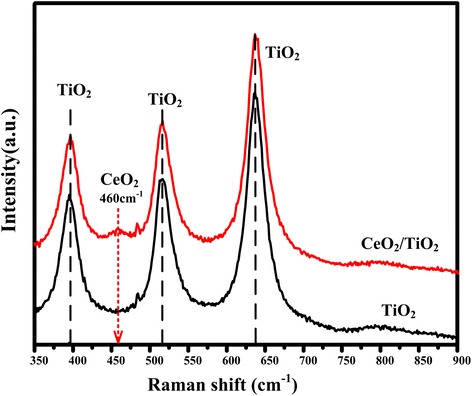
Raman spectra of pure TiO_2_ NTs and CeO_2_/TiO_2_ heterojunction, indicating CeO_2_ NPs were successfully loaded into the TiO_2_ NTAs

In order to further investigate the obtained films, Raman spectroscopy was used to analyze the properties of the CeO_2_-loaded TiO_2_ film. Figure 7 shows two typical Raman spectra of the pure anodic TiO_2_ film and the CeO_2_/TiO_2_ heterojunction film which is prepared in the 1 mol/L Ce(NO_3_)_3_ solution. Peaks located at around 400, 530, and 645 cm^−1^ could be clearly observed, which could be attributed to anatase TiO_2_ phase. Along with these characteristic peaks of anatase TiO_2_, there is a new peak at about 460 cm^−1^ that could be observed for the CeO_2_/TiO_2_ films. According to the Raman-active mode, this peak could be ascribed to the cubic phase of CeO_2_ [39]. The Raman spectra results also confirm that the CeO_2_/TiO_2_ heterojunction was successfully prepared.

### Mechanism of the CeO_2_/TiO_2_ Heterojunction Formation

**Fig. 8:**
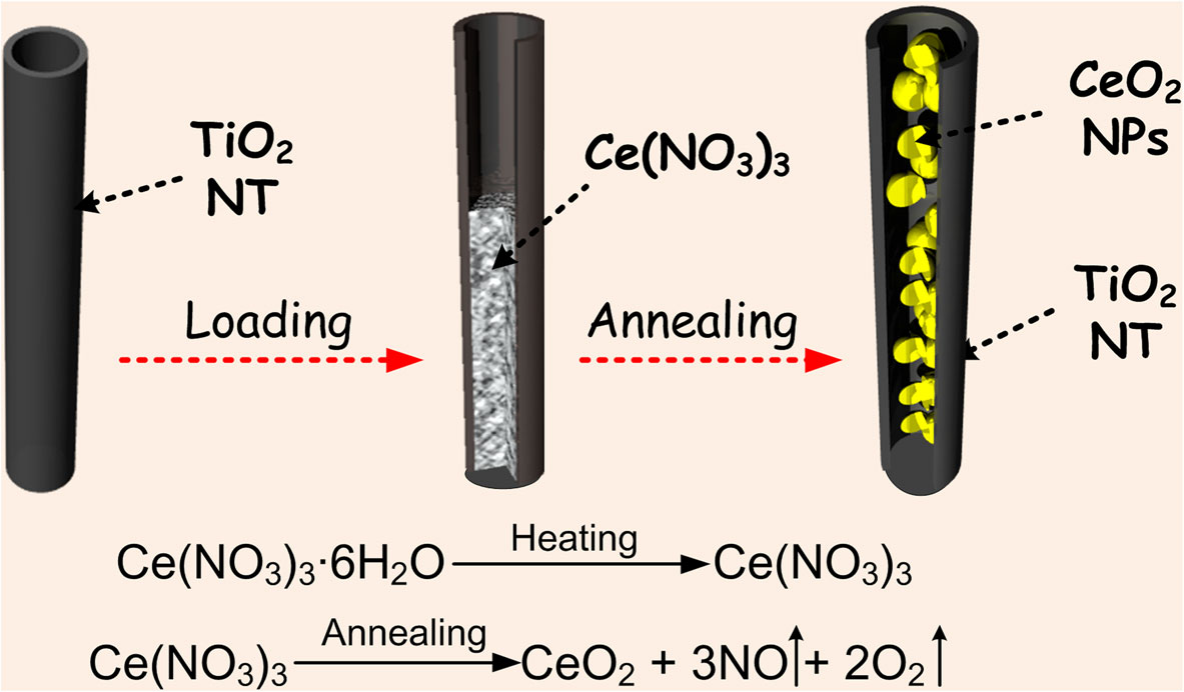
Schematic synthesis diagram of the CeO_2_/TiO_2_ heterojunctions and involved chemical equations

According to the reported studies, the most common used method for preparing CeO_2_/TiO_2_ heterojunction is the sol-gel method or the secondary redox method [40]. In order to obtain the CeO_2_/TiO_2_ heterojunction in a very simple procedure with low cost, in this paper, the preparation of CeO_2_/TiO_2_ heterojunction is achieved by filling TiO_2_ NT nano-container with Ce(NO_3_)_3_ solution and then thermal decomposition of Ce(NO_3_)_3_. The high temperature breaks the chemical bonds of Ce(NO_3_)_3_ molecules, and the decomposed Ce, O, and N atoms then reform into CeO_2_ NPs and NO/O_2_. This process is schematically shown as Fig. 8. Firstly, the Ce(NO_3_)_3_ aqueous solution with different concentrations were filled into the TiO_2_ NT nano-container. Then, the film were baked at 70 °C for 1 h, during which Ce(NO_3_)_3_ will be deposited from water in the form of Ce(NO_3_)_3_·6H_2_O and finally change into Ce(NO_3_)_3_ loaded inside those TiO_2_ NT nano-container. Then, the Ce(NO_3_)_3_-loaded TiO_2_ NT films were annealed at a high temperature of 450 °C for 2 h. Under high temperature conditions, the chemical bonds in the Ce(NO_3_)_3_ molecule will be broken and recombine, resulting in the generation of CeO_2_ NPs inside the TiO_2_ NTs. Two involved chemical reaction are expressed as following eq. (1) and (2):

1 $$ \mathrm{Ce}{\left({\mathrm{NO}}_3\right)}_3\bullet 6{\mathrm{H}}_2\mathrm{O}\to \mathrm{Ce}{\left({\mathrm{NO}}_3\right)}_3 $$

2 $$ \mathrm{Ce}{\left({\mathrm{NO}}_3\right)}_3\to {\mathrm{CeO}}_2\kern0.5em +\mathrm{NO}\uparrow \kern0.5em +{\mathrm{O}}_2\uparrow $$

In short, we have shown a facile method using TiO_2_ NT nano-container to load Ce(NO_3_)_3_ to prepare CeO_2_/TiO_2_ heterojunction films. Ce(NO_3_)_3_ thermal decomposition inside each individual anodic TiO_2_ NTs allows for a good formation and distribution of the CeO_2_ NPs. CeO_2_/TiO_2_ heterojunction films have lots of potential applications. In the field of photocatalysis, it can be used to degrade water pollution, because CeO_2_ can inhibit the rapid electron-hole recombination of TiO_2_ and the heterojunction films can adsorb organic pollutants efficiently. In the field of the photocatalytic hydrogen production and the improvement of TiO_2_ oxygen sensor, CeO_2_ NPs/TiO_2_ NTA films can also be used well.

## Conclusions

Self-organized TiO_2_ NT arrays were prepared through an electrochemistry process, and they were taken as nano-containers to load CeO_2_ raw materials. After thermal treatment, well-distributed CeO_2_ NPs were successfully obtained and loaded onto TiO_2_ NT arrays, forming CeO_2_/TiO_2_ heterojunction films. The formation of cubic CeO_2_ and anatase TiO_2_ were confirmed by XRD. Microscopic morphologies of different CeO_2_/TiO_2_ heterojunction are characterized by SEM, which shows the CeO_2_ NPs were tightly deposited both around the tube and inside the inner wall of the TiO_2_ NT arrays. The successful preparation of CeO_2_/TiO_2_ heterojunction films were also confirmed by EDS and Raman spectra. In summary, this study provides a simple method to prepare CeO_2_/TiO_2_ heterojunction films with good morphology, heterogeneous stability, and low cost, which would be promising for environmental and energy-related applications.

## Abbreviations

EDS: Energy-dispersive spectrometry
NT: Nanotube
SEM: Scanning electron microscopy
XRD: X-ray diffraction
